# Integrative Omics Analyses Reveal the Effects of Copper Ions on Salvianolic Acid Biosynthesis

**DOI:** 10.3389/fpls.2021.746117

**Published:** 2021-10-21

**Authors:** Yaping Xiang, Xiaoxiao Wang, Wei Song, Jinfa Du, Xiaojian Yin

**Affiliations:** ^1^State Key Laboratory of Natural Medicines, Department of Pharmacognosy, Institute of Pharmaceutical Science, China Pharmaceutical University, Nanjing, China; ^2^School of Traditional Chinese Pharmacy, China Pharmaceutical University, Nanjing, China

**Keywords:** copper ion treatment, metabolomics, transcriptomic, *Salvia miltiorrhiza*, salvianolic acids

## Abstract

Salvianolic acids, a group of secondary metabolites produced by *Salvia miltiorrhiza*, are widely used for treating cerebrovascular diseases. Copper is recognized as a necessary microelement and plays an essential role in plant growth. At present, the effect of copper on the biosynthesis of SalAs is unknown. Here, an integrated metabolomic and transcriptomic approach, coupled with biochemical analyses, was employed to dissect the mechanisms by which copper ions induced the biosynthesis of SalAs. In this study, we identified that a low concentration (5 μM) of copper ions could promote growth of *S. miltiorrhiza* and the biosynthesis of SalAs. Results of the metabolomics analysis showed that 160 metabolites (90 increased and 70 decreased) were significantly changed in *S. miltiorrhiza* treated with low concentration of copper ions. The differential metabolites were mainly involved in amino acid metabolism, the pentose phosphate pathway, and carbon fixation in photosynthetic organisms. The contents of chlorophyll *a*, chlorophyll *b*, and total chlorophyll were significantly increased in leaves of low concentration of copper-treated *S. miltiorrhiza* plants. Importantly, core SalA biosynthetic genes (*laccases* and *rosmarinic acid synthase*), SalA biosynthesis-related transcription factors (*MYBs* and *zinc finger CCCH domain-containing protein 33*), and chloroplast proteins-encoding genes (*blue copper protein* and *chlorophyll-binding protein*) were upregulated in the treated samples as indicated by a comprehensive transcriptomic analysis. Bioinformatics and enzyme activity analyses showed that laccase 20 contained copper-binding motifs, and its activity in low concentration of copper ions-treated *S. miltiorrhiza* was much higher than that in the control. Our results demonstrate that enhancement of copper ions of the accumulation of SalAs might be through regulating laccase 20, MYBs, and zinc finger transcription factors, and photosynthetic genes.

## Introduction

Copper, a necessary microelement in plants, plays an essential role in various physiological activities during plant growth and development, such as cell wall metabolism, hormone signaling, photosynthesis, and redox reactions (Choudhary et al., 2012; De Caroli et al., 2020; Ding et al., 2020; Saleem et al., 2020). Copper is a redox-active transition metal, and two valence states (Cu^2+^ and Cu^+^) exist under physiological conditions. Copper can work as a metal cofactor in metalloproteins involved in electron transport and oxidative stress response (Quist et al., 2017). In chloroplasts, copper is a constituent of plastocyanin (Pc), the most abundant copper protein in plant chloroplasts, which acts as an electron carrier in primary photosynthetic reactions (Droppa et al., 1984). Copper also works as a constituent of Cu/Zn-superoxide dismutase (Cu/Zn-SOD), localized in the stroma that protects against reactive oxygen species (Yruela, 2009). Due to these characters, copper ion plays an important role in regulating plant physiology.

Although copper is indispensable in plant development, most studies have reported that excessive copper causes toxicity to plants *via* the production of reactive oxygen species (ROS) that can oxidize biological macromolecules, such as lipids and nucleic acids, and cause enzyme inactivation (Andre et al., 2010). It has been reported that excessive copper causes rice cell death by increasing the ROS level in rice radicles (Zhang et al., 2017). However, a low dose of copper can enhance enzyme activity to increase the production of plant secondary metabolites (Ibrahim et al., 2017). Treatment of Arabidopsis seedlings with different concentrations of copper showed that the right amount of copper increased the meristem size of the seedlings (Song et al., 2017). Although pieces of research on copper have reported its role in several biological functions involving cytochrome c oxidase, plastocyanin, and ethylene receptors (Burkhead et al., 2009; Song et al., 2017), the majority of previous studies have not focused on whether copper ions are involved in plant secondary metabolism. Therefore, studies are needed to improve our understanding of the role of copper in plant secondary metabolism.

*Salvia miltiorrhiza*, a traditional Chinese herb, is widely used in the treatment of cardiovascular diseases. Water-soluble phenolic acids, such as salvianolic acid A, salvianolic acid B (Sal B), and rosmarinic acid (RA), are the essential active compounds in the roots of *S. miltiorrhiza* (Huang et al., 2019). There are two biosynthetic routes for phenolic acid in plants: the tyrosine-derived and phenylpropanoid pathways. In the tyrosine-derived pathway, tyrosine is converted to 4-hydroxyphenyllactate under the action of various enzymes, including tyrosine aminotransferase and 4-hydroxyphenylpyruvate reductase. In the phenylpropanoid pathway, phenylalanine is converted to 4-coumaroyl-CoA under the action of various enzymes, including phenylalanine ammonia-lyase, cinnamic acid 4-hydroxylase, and 4-coumarate: CoA ligase. Rosmarinic acid synthase catalyzes the transformation of 4-coumaroyl-CoA and 4-hydroxyphenyllactate to rosmarinic acid (Ma et al., 2013; Shi et al., 2019). The molecular mechanism by which rosmarinic acid converts to salvianolic acid B is still not completely understood. Recently, laccase has been found to catalyze the conversion of rosmarinic acid to salvianolic acid E, which is then transformed into salvianolic acid B and other compounds (Li et al., 2019a; Wang et al., 2019). Furthermore, many transcription factors also regulate the biosynthesis of phenolic acids (Xing et al., 2018; Sun et al., 2019; Deng et al., 2020a; Yin et al., 2020).

As ceruploplasmin oxidases, laccases are widely distributed in all kinds of organisms, including bacteria, fungi, insects, and plants. Laccases belong to the multicopper oxidases family, and four copper ions existed in laccase (Hoegger et al., 2006). Laccases contain three types of catalytic sites (T1, T2, and T3), and T1 site is capable of oxidating substrates, while T2/3 sites are responsible for the consumption of oxygen and the generation of water (Solomon et al., 1996; Riva, 2006). T1 site containing one blue copper ion is mainly responsible for taking the electrons from the substrate after the laccase protein binds to the substrate. The remained three copper ions existed in T2 and T3 sites; they, together, form into the T2/T3 trinuclear center (copper cluster) (Patel et al., 2019). After the T1-copper ion obtains electrons, the electrons will be transferred to the T2/T3 trinuclear center *via* the amino acid bridge, and oxygen molecules obtained from the environment are reduced to produce water (Jones et al., 2015). In a word, the process of laccase catalyzes the oxidation by taking electrons from the substrate and transferring them to the corresponding domains and reacts with oxygen in the environment to form water. Meanwhile, the substrate loses electrons and becomes free radicals. To date, a large number of laccases have been identified and reported to exert their function in plant lignin biosynthesis, cell wall biosynthesis, and flavonoid biosynthesis (Turlapati et al., 2011). In *Arabidopsis*, 17 laccases have been annotated, which are clustered into six distinct clades (McCaig et al., 2005; Turlapati et al., 2011). In *S. miltiorrhiza*, laccases were also systemically investigated and demonstrated to play a great role in plant growth, development, and secondary metabolites biosynthesis (Zhou et al., 2018; Li et al., 2019a).

When a plant encounters adverse stress, the plant's own stress resistance mechanism will be activated and related genes will control the process of protein synthesis to protect themselves against adversity. Here, metabolomics and transcriptomics approaches were applied to reveal how copper affects the biosynthesis of phenolic acids in *S. miltiorrhiza*. The genes that respond to copper and the genes in the biosynthetic pathway of salvanoic acids (SalAs) were analyzed. Our findings are of great significance in understanding the underlying mechanism of the induction of the biosynthesis of SalAs under copper treatment and provide potential approach to improve SalA content in *S. miltiorrhiza*.

## Methods

### Plant Materials and Treatment

*Salvia miltiorrhiza* was cultivated at 25°C with a 16-h-light/8-h-dark cycle in MS basal medium containing 3% sugar and 8% agar. CuSO_4_·5H_2_O (Sinopharm Chemical Reagent Company., Ltd, China) was dissolved in distilled water to make concentrations of 5, 25, and 100 mM. All the prepared CuSO_4_·5H_2_O were sterilized through 0.22-μm filters and then added to MS basal medium to a final concentration of 5, 25, or 100 μM. *S. miltiorrhiza* was treated with different concentrations of CuSO_4_·5H_2_O for 5 days, and then plant matter was collected for the following experiments.

### Phenotype and Fresh Weight

After 5 days of treatment with different concentrations of CuSO4·5H_2_O, a photo *of S. miltiorrhiza* planted in a culture flask was taken. To measure fresh weight, the whole plant of *S. miltiorrhiza* was taken out from the culture flask, washing away culture medicum, and measured with electronic balance. To accurately reflect fresh weight changes, at least four plants were used each time, and average weight was calculated.

### High-Performance Liquid Chromatography (HPLC)

To extract phenolic acids, fresh roots of copper-treated or non-treated *S. miltiorrhiza* were collected from a culture flask, dried at 55°C until the weight remained constant, and then grounded into powder. Each sample of 0.1 g was added to 1-ml methanol: water (70: 30, v v-1), followed by 1-h ultrasound and centrifugation at 12,000 rpm for 20 min. The supernatant was then removed and filtered through a 0.22-μm filter. HPLC was performed on an Agilent 1290 with a DAD detector (Agilent Technologies, USA), equipped with an Agilent ODS-SP 5-μm 4.6 mm × 250 mm column. The mobile phase was H_2_O (0.1% formic acid, A) and acetonitrile (B). The HPLC program was set to the following line gradient: 10–25% of B for 0–10 min, 25–27% of B for 10–15 min, 27–55% of B for 15–20 min, 55–95% of B for 20–21 min, held 5 min, 95–10% of B for 26–27 min, and 10% of B for 27–30 min. The flow rate was 1 ml/min, the column temperature was set to 35°C, sample injection volume was 10 μl, and the wavelength was 280 nm. Sal B and RA standards (Shanghai Yuanye Bio-Technology Co., Ltd, China) were configured as a mixed standard solution with a concentration of 0.25 mg/ml. The standard curve method was used to calculate the content of Sal B and RA. The experiment was performed with four repetitions, and data were expressed as mean ± SD.

### Ultra-Performance Liquid Chromatography-Quadrupole-Time of Flight-Mass Spectrometry (UPLC-QTOF MS)

For metabolomics analysis, the same samples were analyzed by LC-MS using an Agilent 6545A Q/TOF mass spectrometer equipped with an electrospray ion (ESI) source. In the negative ion mode, the following conditions were used: a drying N2 gas flow rate, 8 L/min; temperature, 320°C; nebulizer, 35 psig; capillary, 3,000 V; skimmer, 65 V; OCT RFV, 750 V. Mass spectra were recorded in a full-scan mode from 100 to 1,200 m/z range. Agilent 1290 Infinity Ultra-performance liquid chromatography (UPLC, Agilent technologies, USA), equipped with an Agilent ODS-SP 5 μm 4.6 mm × 250 mm column, was used to affect the separations. The mobile phase consisted of acetonitrile and 0.03% acetic acid (mobile phase A) and H2O with 0.03% acetic acid (mobile phase B), which were eluted as follows: 10–13% of B at 0–8 min, 13–23% of B at 8–13 min, 23% of B at 25 min, 23–27% of B at 25–45 min, 27–36% of B at 45–54 min, 36–70% of B at 54–65 min, 70–73% of B at 65–75 min, 73–93% of B at 75–85 min, 93–100% of B at 85–87 min, and then held for 5 min. The flow rate was 0.5 ml/min; the column temperature was set at 35°C, sample injection volume was 5 μl, and post-run time was 12 min.

For data analysis, raw LC-MS data were converted to mzData format using DA reprocessor software (Agilent), and the XCMS package in R was used to perform peak finding, filtering, and alignment. Metabioananlyst 4.0 (https://www.metaboanalyst.ca) was used to obtain normalized data. Metabolites with *P* < 0.05 and fold change (copper treatment/blank) >1.5 or < 0.5 were regarded as differential metabolites. Metlin database, Human Metabolome database, and our in-house laboratory database were used to identify metabolites. The experiment was performed with four repetitions, and data were expressed as mean ± SD.

### Global Transcriptomic Analysis

Total RNA was isolated from fresh root of copper-treated or non-treated *S. miltiorrhiza* using RNeasy Plus Kit with the genomic DNA removal step. The concentration and the quality of extracted RNA were evaluated. cDNA library construction and sequencing were performed by the Biomarker Technologies Corporation (Beijing, China). The raw sequence data were filtered to obtain clean data, which were then compared with the *S. miltiorrhiza* reference genome using HISAT2 software (https://ccb.jhu.edu/software/hisat2/manual.shtml). Fragments Per Kilobase of transcript per Million fragments mapped (FPKM) was used to calculate gene expression. Differential gene expression analysis used edgeR (Robinson et al., 2010), and genes with *p* < 0.05 and fold change >1.5 or <0.5 are regarded as differentially expressed genes (DEGs). The DEGs were classified into functional categories by blasting against the clusters of orthologous groups against the eukaryotic complete genomes (KOG) database (http://www.ncbi.nlm.gov/KOG) and the gene ontology (GO) database (http://geneontology.org/). The experiment was performed with three repetitions, and data were expressed as mean ± SD.

### Total RNA Extraction and Reverse Transcription-Quantitative PCR (RT-qPCR)

Total RNA extraction was achieved using a plant total RNA extraction kit (Tiangene Biotech Co., Ltd., China). Briefly, a 0.5-g fresh *S. miltiorrhiza* root was grounded into powder with liquid nitrogen, and extraction was carried out according to the instructions. RNA reverse transcription (Hieff™ First Strand cDNA Synthesis Super Mix, Yeasen, China) and gene quantification (Hieff™ qPCR SYBR Green Master Mix kit, Yeasen, China) were performed following the protocols of the manufacturer. The qPCR conditions were as follows: 95°C for 5 min, followed by 40 cycles of 95°C for 10 s, 60°C for 30 s, and 70°C for 90 s. mRNA expression levels were calculated using the ^2−ΔΔ^Ct method and presented as a ratio to β-actin. Primer sequences are shown in Supplementary Table 4.

### Quantification of Laccase Enzyme Activity

A Laccase Activity Detection kit (Solarbio Life Sciences, Beijing, China) was used to detect laccase activity. To detect laccase activity, a 0.1-g fresh *S. miltiorrhiza* root was added to a 1-ml extraction buffer, the mixture was homogenized on ice, and then centrifuged at 12,000 rpm for 20 min at 4°C. The supernatant was then moved to a new centrifuge tube, and the absorbance was measured at 562 nm to determine protein concentration of supernatant using a BCA kit (Good Laboratory Practice Bioscience, Montclair, CA, USA). Subsequently, 150 μL of supernatant was mixed with 850 μL of a working buffer and heated in an oven at 45°C for 3 min. As negative control, 150 μL of an extraction buffer was mixed with 850 μL of the working buffer and heated in an oven at 45°C for 3 min. Then, the produced ABTS radical was determined through measuring mixture absorbance at 420 nm. Enzyme activity was calculated through measuring ABTS radical content change according to the instructions of the manufacturer.

### Phylogenetic Construction of Laccase

The whole genome sequence and protein data from *S. miltiorrhiza* (BioProject: PRJNA287594) were downloaded from the Genome Warehouse in the BIG Data Center (http://gigadb.org/dataset/100164). The whole genome data and protein data of *Arabidopsis thaliana* were downloaded from the *Arabidopsis thaliana* information resource website (TAIR, https://www.arabidopsis.org/). In order to select all the laccases in the genomes of *Salvia miltiorrhiza* and *Arabidopsis thaliana*, we downloaded the unique HMM model (the hidden Markov model) of laccase from the Pfam database. HMMER 3.0 software with hmmsearch was used to search for laccase-conserved domain models, including PF00394, PF07731, and PF07732, in the protein database of *S. miltiorrhiza* and *Arabidopsis thaliana*. A phylogenetic tree was constructed using the maximum likelihood (ML) method with bootstraps in MEGA X software.

### Correlation Analysis

FPKM values of genes in copper ion-treated and non-treated groups were used to calculate correlation coefficient. Gene co-expression analysis between the candidate transcription factors and the key genes was performed by the mean of the Pearson correlation test using IBM SPSS Statistics 20. Data are shown as the mean ± SD (*n* = 3) (^*^p < 0.05 and ^**^p < 0.01). The correlation coefficient >0.8 was considered to be co-expressed, and the coexpression network was visualized using Cytoscape.

### Copper-Response Element Predication in Laccases and Transcription Factors

The promoter sequences of analyzed genes (from 2,000 bp, relative to a translation start site) were extracted using gff file of *S. miltiorrhiza* genome. Core motif of copper-response element (GTAC) (Quinn et al., 2000; Kropat et al., 2005) and metal response-element (TGCxCxC) (Murphy et al., 1999) were predicted in extracted 2,000 bp promoter sequences of laccases and transcription factors using the Plantcare database (Lescot et al., 2002) and visualized by TB tools (Chen et al., 2020). In addition, transcription factors-binding motifs were also predicated in extracted 2,000 bp promoter sequences of laccases.

## Results

### A Low Concentration of Copper Ions Promoted Plant Growth

**Figure 1:**
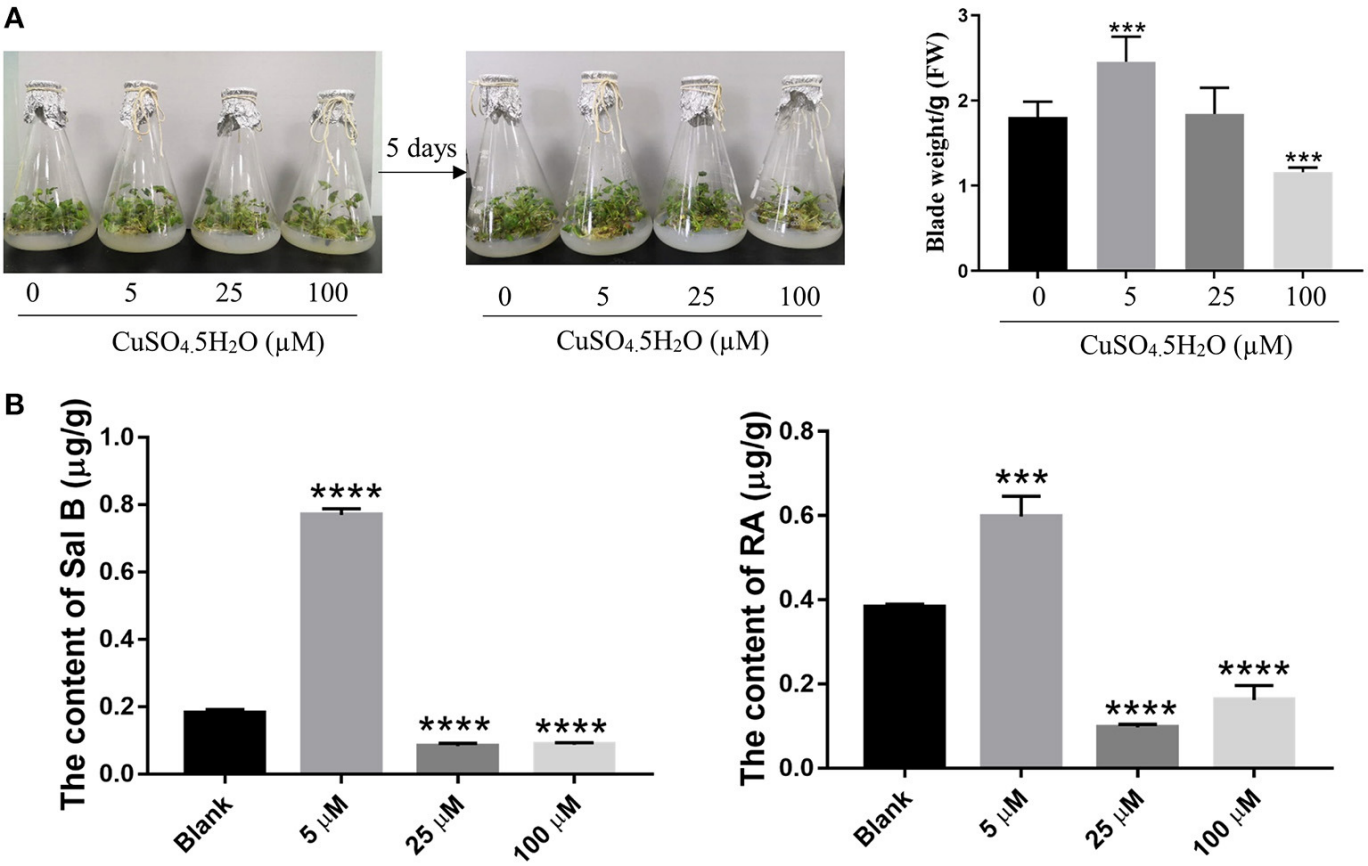
Effect of copper ion treatment on the growth of *Salvia miltiorrhiza* and biosynthesis of salvinolic acids (SalAs). Two-month-old *S. miltiorrhiza* was treated with different concentrations of CuSO_4_.5H_2_O (0, 5, 25, and 100 μM) for 5 days. **(A)** Phenotype change and weight change of *S. miltiorrhiza* were recorded before and after the treatment. **(B)** The content of salvianolic acid B and rosemarinic acid in untreated and treated *S. miltiorrhiza* using high-performance liquid chromatography (HPLC) with standards. Data represent the mean ± SD of three independent biological replicates. Significant differences between the treatment and control samples were determined using a Student's *T*-test (***P < 0.001 and ****P < 0.0001).

To explore the effect of copper on the growth of *S. miltiorrhiza* plants, we exposed the plants to predetermined copper concentrations (0, 5, 25, and 100 μM) and determined the phenotypic changes thereafter. After 5 days of cultivation, the *S. miltiorrhiza* plants wilted under treatments with a greater copper concentration. Compared with the 0 and 5 μM treatments, *S. miltiorrhiza* plants grew slowly and the leaves withered and yellowed in the 25- and 100-μM treatments (Figure 1A). It is noteworthy that low concentration of copper (5 μM) significantly promoted plant growth compared with the blank (0 μM) group (Figure 1A). Thus, 5 μM of copper was optimum for the growth of the *S. miltiorrhiza* plants.

### A Low Concentration of Copper Ions Promoted the Accumulation of Sal B and RA

As the major SalAs, Sal B, and RA are important active secondary metabolites in *S. miltiorrhiza*. To ascertain the most suitable concentration of copper that promotes SalAs biosyntheses, we used HPLC to determine the contents of Sal B and RA in the copper-stressed groups and the blank group. The low concentration of copper treatment increased Sal B by an average of 4.27-fold compared with the control, but the medium and high concentrations of copper treatments decreased Sal B by 2.16- and 2.06-fold, respectively, compared with the control (Figure 1B and Supplementary Figure 1). Compared with the blank group, low concentration of copper increased RA content 1.55-fold while medium and high concentrations of copper decreased its amount 4.22- and 2.37-fold, respectively (Figure 1B and Supplementary Figure 1). Based on these findings, it is evident that low concentration of copper promotes the biosyntheses of Sal B and RA.

### A Low Concentration of Copper Increases the Content of SalAs

**Figure 2:**
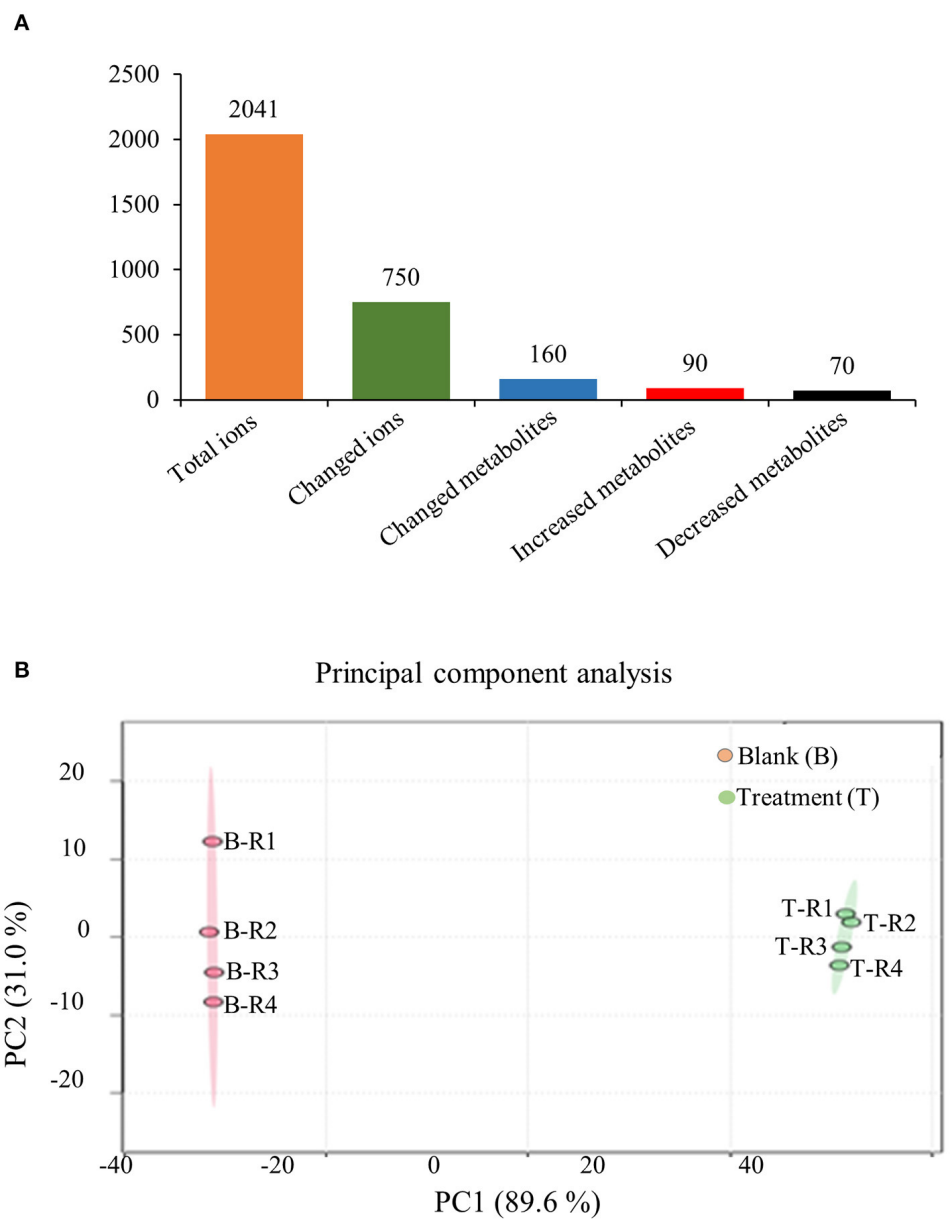
Metabolomic analysis of low concentration of copper ion (LCCI) on the biosynthesis of salvinolic acids (SalAs) in *Salvia miltiorrhiza*. Two-month-old *S. miltiorrhiza* was treated with CuSO_4_.5H_2_O (5 μM) for 5 days. Metabolites were extracted from roots and subjected to metabolomics analysis by untargeted ultra-performance liquid chromatography-quadrupole-time of flight mass spectrometry (UPLC-Q/TOF-MS). The total ions were counted within 90 min. **(A)** The statistical number of identified ions, metabolites, and significantly changed metabolites is listed. **(B)** Principal component analysis (PCA) and a volcano plot based on identified ions in control and treated samples. To characterize their ion profiles, the identified ions were estimated using a principal component analysis. The significantly changed ions are depicted with a volcano plot. Red dots indicate significantly changed ions.

**Figure 3:**
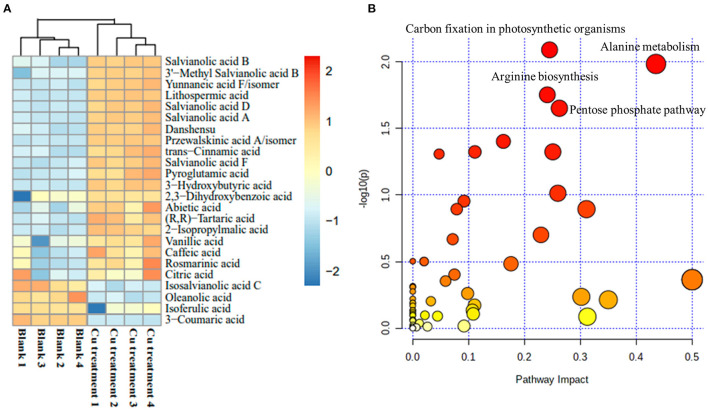
Change of identified salvinolic acids (SalAs). Two-month-old *S. miltiorrhiza* was treated with CuSO_4_.5H_2_O (5 μM) for 5 days. **(A)** The changes of identified SalAs in control and treated samples were analyzed using a Cluster analysis. **(B)** Pathway enrichment analysis of significantly changed metabolites was performed using the KEGG database.

To further confirm the holistic effect of low copper on the accumulation of SalAs in *S. miltiorrhiza*, the plant samples exposed to low concentration of copper and the blank group were analyzed using UPLC-QTOF-MS. In the negative iron model, the total ion chromatogram was different between the control and the low copper treatment (Supplementary Figure 2). A total of 2,041 ions were detected (Figure 2A). Using the selection criteria of change ratio >1.5 (significant increase; *P* < 0.05) or < 0.5 (significant decrease; *P* < 0.05), 90 increased and 70 decreased compounds were detected. The principal component analysis (PCA) analysis showed that the copper stress treatment and control clearly clustered into two categories (Figure 2B). Among the different metabolites, 13 SalAs, including Sal B, RA, lithospermic acid, salvianolic acid D, salvianolic acid A, and danshensu, were significantly increased in the low concentration of copper treatment compared with the control (Figure 3A), indicating that low copper stress can positively regulate the synthesis of SalAs. The pathway enrichment analysis of differential metabolites showed that copper ions had significant effects on amino acid synthesis and metabolism, the pentose phosphate pathway, and carbon fixation in photosynthesis in *S. miltiorrhiza* (Figure 3B).

### Transcriptomics Analysis to Identify Genes That Responded to Copper Treatment in *S. miltiorrhiza*

**Figure 4:**
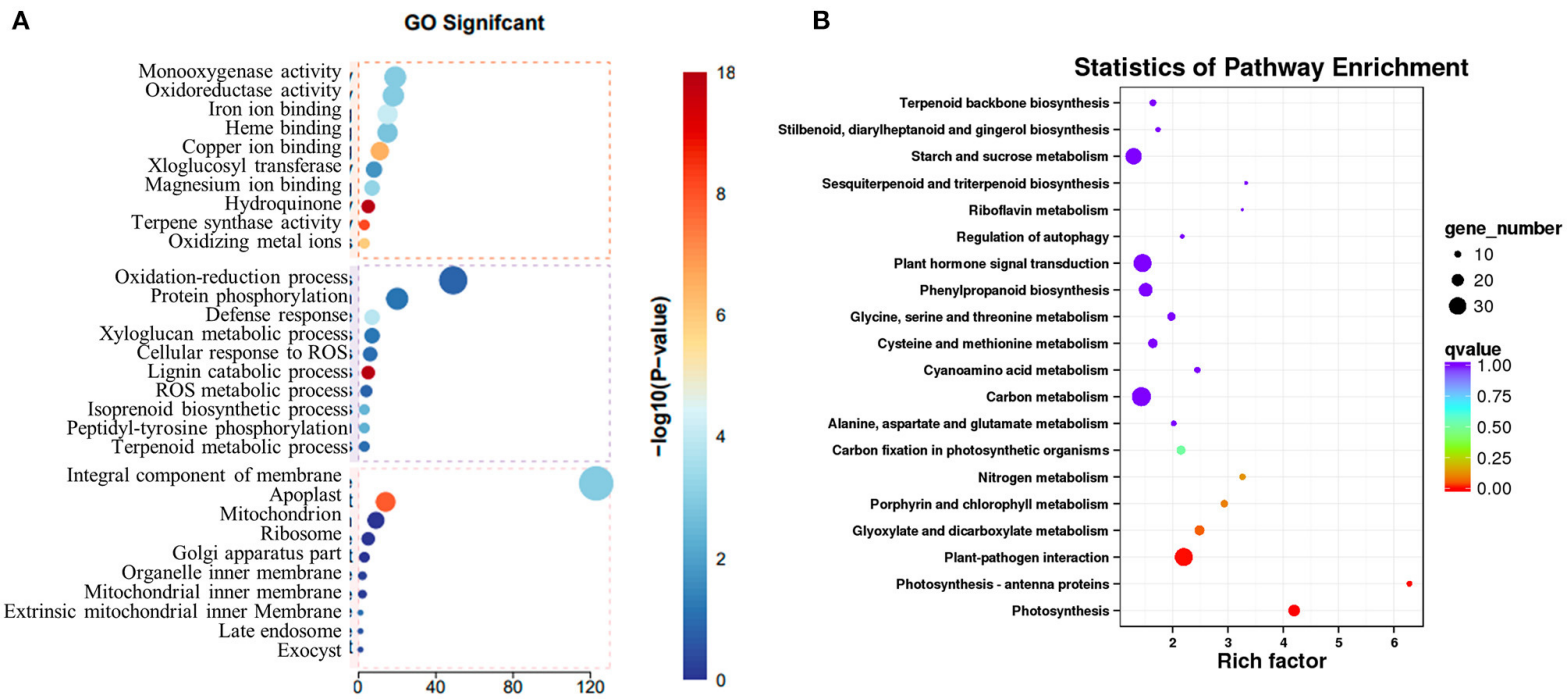
Transcriptomic analysis of *Salvia miltiorrhiza* with low concentration copper treatment. **(A)** Two-month-old *S. miltiorrhiza* was treated with CuSO4.5H_2_O (5 μM) for 5 days, following which mRNA was extracted from roots and subjected to transcriptomic analysis. **(A)** Differentially expressed genes were identified by comparing gene expression in copper treated *vs*. untreated samples. Function of identified significantly changed genes was analyzed using the gene ontology (GO) database. **(B)** Pathway mapping of significantly changed genes was performed using the Kyoto Encyclopedia of Genes and Genomes database (http://www.genome.jp/kegg/).

To explore how the low concentration of copper treatment regulated the biosynthesis of SalAs in *S. miltiorrhiza*, a transcriptome analysis was performed between the control and low copper treatments. Using the selection criteria of the change ratio *P* < 0.05 and fold change >1.5 and <0.5, a total of 418 differential genes were obtained, of which 207 were upregulated and 211 were downregulated in the low copper-treated group compared with the blank group (Supplementary Table 2). Gene Ontology (GO) analysis showed that these significantly changed genes were mainly involved in copper ion binding, oxidoreductase, and ROS metabolism (Figure 4A). Pathway enrichment analysis showed that the differentially expressed genes are related to tyrosine and phenylalanine metabolism, chlorophyll biosynthesis, plant pathogene interaction, photosynthesis, and plant hormone signal transduction (Figure 4B).

**Figure 5:**
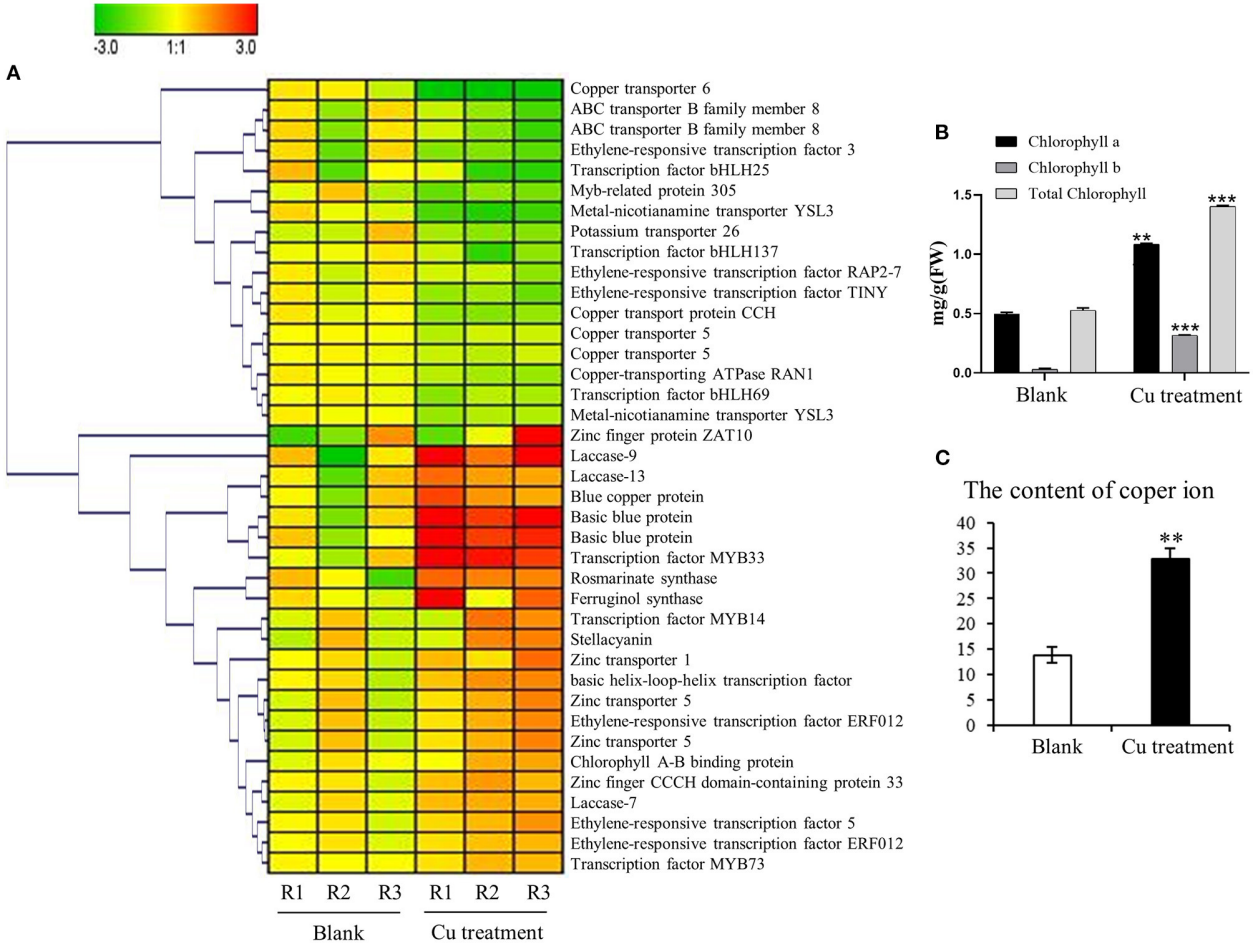
Impact of the copper treatment on biosynthesis of salvinolic acids (SalAs), chlorophyll metabolism, and ion transport. **(A)** The change tendency of differentially expressed genes involved in the biosynthesis of SalAs, chlorophyll metabolism, and ion transport was analyzed based on their expression levels in untreated and treated samples. **(B)** The content of chlorophyll *a*, chlorophyll *b*, and total chlorophyll in untreated and treated samples was determined. **(C)** The content of copper ions in untreated and treated samples was measured. Data represent the mean ± SD of three independent biological replicates. Significant differences between treatment and control samples were determined using a Student's *T*-test (**P < 0.01, ***P < 0.001).

A further gene expression pattern indicated that SalAs biosynthesis-related genes (*laccase 20, laccase 13, laccase 7*, and *rosmarinate synthase*), photosynthesis-related genes (*blue copper protein, basic blue protein*, and *chlorophyll A-B*-*binding protein*), and *zinc transporter 5* were upregulated in the copper-treated group compared with the blank group (Figure 5A and Table 1). In contrast, copper transport-related genes, such as *copper transporter 6, copper transporter 5, copper transport protein CCH*, and *copper-transporting ATPase RAN1*, were downregulated in the copper-treated group (Figure 5A and Table 1). In addition, transcription factors, such as *ethylene-responsive transcription factor 5, ERF012, ethylene-responsive transcription factor RAP2-7, ethylene-responsive transcription factor TINY, transcription factor MYB14, MYB33, MYB73, basic helix-loop-helix transcription factor*, and *transcription factor bHLH69*, were responsive to low copper treatment (Figure 5A and Table 1).

**Table 1:** Key genes involved in salvianolic acid biosynthesis that showed a significantly change in abundance in leaves and roots of Salvia miltiorrhiza in response to treatment with a low concentration of copper ions.

**Gene ID**	**Description**	**Ratio**	***P*-value**
scaffold9670.3	Laccase 20	13.1784	0.0000
scaffold1005.5	Zinc finger protein ZAT10	12.5554	0.0017
scaffold4128.1	Basic blue protein	8.8272	0.0000
scaffold1723.6	Transcription factor MYB33	9.0328	0.0000
scaffold3548.4	Basic blue protein	6.5314	0.0000
newGene_16720	Ferruginol synthase	4.1724	0.0062
newGene_4005	Rosmarinate synthase	2.9856	0.0026
scaffold7426.5	Blue copper protein	2.9906	0.0006
scaffold9243.42	Laccase 13	2.5341	0.0144
scaffold6895.1	basic helix-loop-helix transcription factor	2.2409	0.0055
scaffold8762.12	Zinc transporter 1	2.1111	0.0096
scaffold696.10	Transcription factor MYB14	2.1066	0.0452
scaffold4780.1	Stellacyanin	2.0829	0.0466
scaffold720.3	Zinc transporter 5	1.9774	0.0255
C221085.11	Zinc transporter 5	1.9465	0.0297
scaffold7135.8	Ethylene-responsive transcription factor ERF012	1.9232	0.0284
scaffold9614.5	Zinc finger CCCH domain-containing protein 33	1.8885	0.0135
scaffold215.22	Laccase 7	1.8639	0.0068
scaffold10670.14	Ethylene-responsive transcription factor 5	1.8451	0.0114
scaffold726.5	Chlorophyll A-B binding protein	1.6674	0.0418
C219717.5	Transcription factor MYB73	1.6080	0.0458
scaffold12295.1	Ethylene-responsive transcription factor ERF012	1.5692	0.0432
scaffold4374.6	Copper transporter 5	0.6246	0.0357
scaffold1463.7	Copper transporter 5	0.6236	0.0404
scaffold5168.12	Ethylene-responsive transcription factor RAP2-7	0.5813	0.0393
scaffold239.4	Copper-transporting ATPase RAN1	0.4883	0.0021
C220963.1	Metal-nicotianamine transporter YSL3	0.4967	0.0018
newGene_9124	Transcription factor bHLH69	0.4776	0.0426
scaffold9212.4	Potassium transporter 26	0.4124	0.0269
scaffold4677.5	Putative ABC transporter B family member 8	0.4269	0.0170
scaffold4677.4	Putative ABC transporter B family member 8	0.4154	0.0254
scaffold3984.1	Transcription factor bHLH137	0.4101	0.0339
scaffold3771.63	Ethylene-responsive transcription factor TINY	0.3941	0.0243
scaffold3068.14	Copper transport protein CCH	0.3959	0.0000
C222313.14	Transcription factor bHLH25	0.4072	0.0314
scaffold8569.11	Myb-related protein 305	0.3426	0.0028
scaffold1219.23	Ethylene-responsive transcription factor 3	0.3299	0.0016
scaffold1040.9	Metal-nicotianamine transporter YSL3	0.1990	0.0000
scaffold4004.3	Copper transporter 6	0.0802	0.0000

### Effect of Copper Ions on the Chlorophyll Content in the Leaves of *S. miltiorrhiza*

To determine whether the gene expression changes corresponded to physiological changes, chlorophyll content and copper content in *S. miltiorrhiza* were measured. Chlorophyll, which plays an extremely important role in photosynthesis, is the main component of chloroplasts in leaves. After the copper ion treatment, we detected the difference in chlorophyll content between the 5-μM copper treatment and the control (Figure 5B). The chlorophyll a, chlorophyll b, and total chlorophyll of *S. miltiorrhiza* in the 5-μM copper treatment were 1.08, 0.31, and 1.39 mg/g, respectively. The chlorophyll a, chlorophyll b, and total chlorophyll of *S. miltiorrhiza* in the control were 0.49, 0.03, and 0.52 mg/g, respectively. Compared with the control, the 5-μM copper treatment increased chlorophyll a, chlorophyll b, and total chlorophyll by 2.19, 9.98, and 2.66 times, respectively. Copper content in the treatment group was also significantly increased compared with the control (Figure 5C). The greater chlorophyll content increased the photosynthetic ability of the copper treatment group, the growth state of the plant was healthier, the ability of the plant to resist adverse environments was increased, and the treatment was conducive to the accumulation of active products in *S. miltiorrhiza*.

### Bioinformatics and Enzyme Activity Analysis of Copper Ion-Induced Laccase

**Figure 6:**
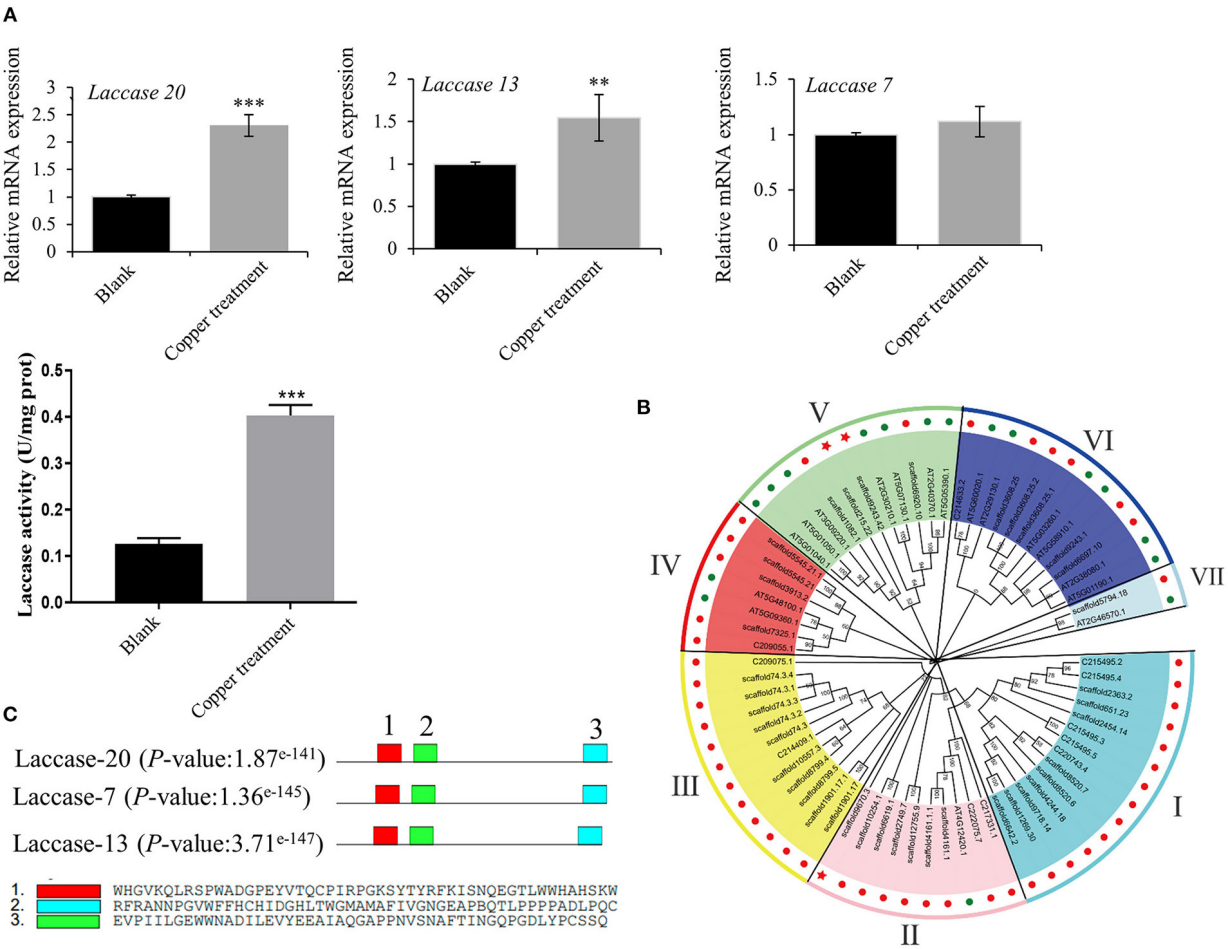
Bioinformatics and enzyme activity analyses of copper ion-induced laccase. **(A)** mRNA expression levels of laccase 20, laccase 13, and laccase 7 in untreated and treated samples were checked by reverse transcription-quantitative PCR (RT-qPCR). Crude proteins were extracted from root of *Salvia miltiorrhiza*, and laccase activity was measured in untreated and treated samples using an ELISA kit. **(B)** A phylogenetic tree of laccases in *Salvia miltiorrhiza* (red dots) and *Arabidopsis thaliana* (green dots) was constructed using the maximum likelihood (ML) method with bootstraps in MEGA X software. Our target laccases (laccase 20, laccase 13, and laccase 7) were marked with a red pentagram. **(C)** A motif analysis of three key laccases was performed using MEME Suite. **P-value < 0.01, ***P-value < 0.001.

Based on the transcriptomics result, we verified the mRNA expression of laccase 20, laccase 13, and laccase 7 using RT-qPCR. The results obtained showed 2.3 and 1.4-fold increases in laccase 20 and laccase 13 expressions, respectively, in the low-copper group relative to the control group. Consequently, laccase enzyme activity increased 3.19-fold in the low-copper group compared with the control group (Figure 6A). These findings suggest the pivotal role of laccase in the synthesis of salvianolic acids in the presence of low copper. To explore the evolutionary relationships among laccases, the sequences of laccases were extracted from *S. miltiorrhiza* and *A. thaliana*, and a phylogenetic tree was constructed using the ML method with bootstraps in MEGA X software (Figure 6B). Laccases in *S. miltiorrhiza* and *Arabidopsis thaliana* were grouped into seven clusters (I, II, III, IV, V, VI, and VII) based on similarity of amino acid sequence. The copper-induced laccases (marked with a red pentagram) were positioned in Cluster II and Cluster V (Figure 6B). Functional domain analysis found that the three key laccases had the same three motifs and conserved domains, which implies that they have functional similarity (Figure 6C).

**Figure 7:**
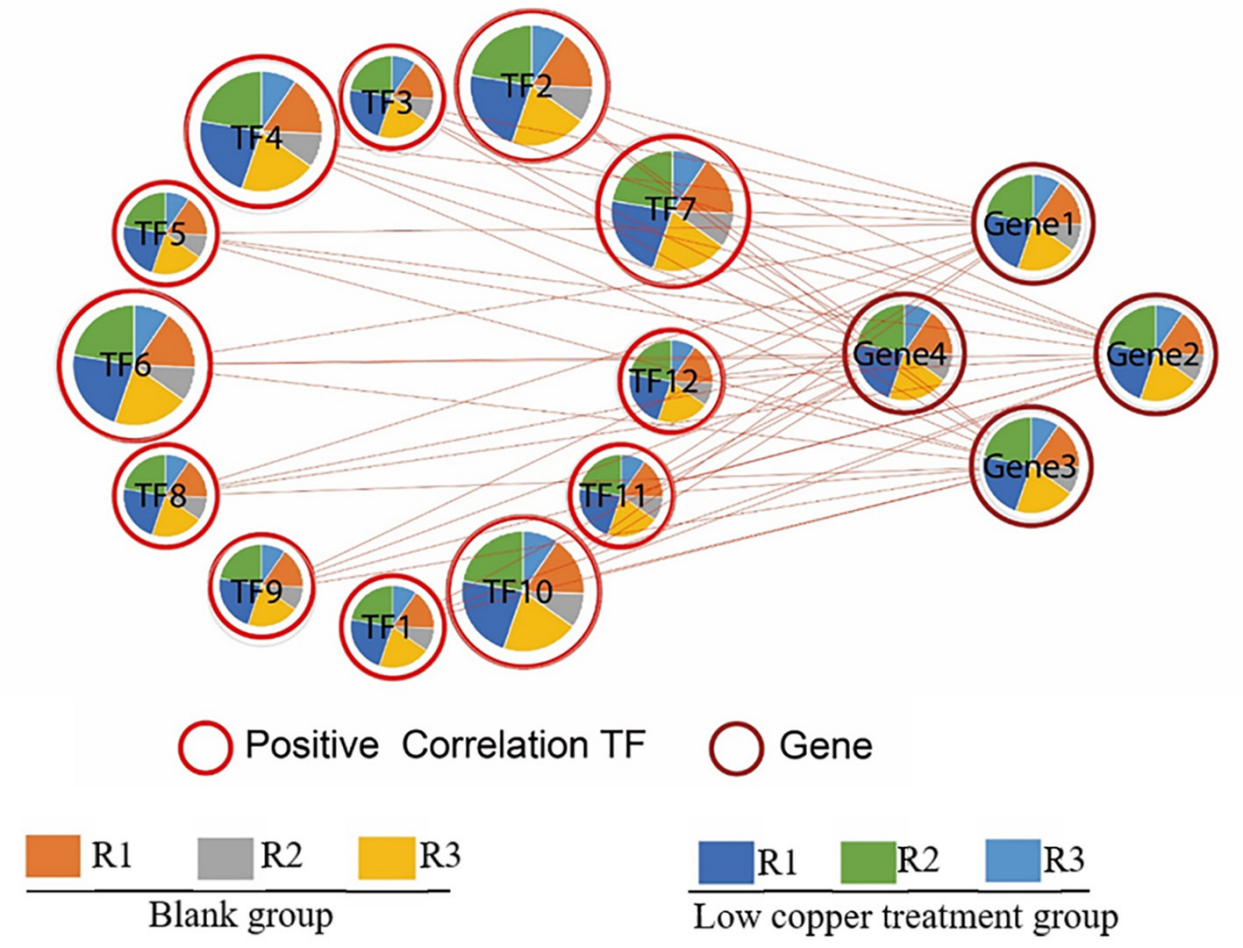
Correlation analysis between significantly changed transcription factors and enzymes related to SalA biosynthesis. The correlation between significantly changed SalA biosynthetic enzymes and the relative expression of candidate transcription factors was analyzed using R software. A Spearman correlation analysis was performed to calculate the correlation coefficient.

To identify the upstream transcription factor involved in the regulation of copper-induced biosynthetic enzymes of SalAs, a coexpression analysis of biosynthetic genes related to SalAs (laccases and rosmarinate synthase) and 12 key transcription factors was carried out. As shown in Figure 7, transcription factors were positively correlated with key candidate genes in the biosynthetic pathways of SalAs. Among them, zinc finger protein ZAT10, Myb family transcription factor PHL5, ethylene-responsive transcription factor 5, and bZIP transcription factor 18 were significantly coexpressed with laccase 20 (gene 3); Zinc finger CCCH domain-containing protein 33/29, zinc finger A20 and AN1 domain-containing stress-associated protein 5, transcription factor MYB73, and ethylene-responsive transcription factor ERF012 were significantly coexpressed with laccase 7 (gene 1); Transcription factor MYB33 was significantly coexpressed with laccase 13 (gene 3) (Supplementary Table 3). To further explore the underlying mechanism by which copper induces expression of laccases and transcription factors, a copper-response element and metal-response elements were searched against 2,000 bp promoter sequences of laccases and transcription factors. As shown in Supplementary Figure 3, a core motif of a copper-response element (GTAC) (Quinn et al., 2000; Kropat et al., 2005) and a metal-response element (TGCxCxC) (Murphy et al., 1999) were identified in extracted 2,000 bp promoter sequences of laccases (laccase 20 and laccase 13) and transcription factors (zinc finger protein ZAT10, Zinc finger CCCH domain-containing protein 33, transcription factor MYB73, and transcription factor MYB33). In addition, MYB transcription factor-binding motifs were also identified in promoter sequences of laccase 20 and laccase 13 (Supplementary Figure 3).

## Discussion

SalAs are important water-soluble compounds in *S. miltiorrhiza*, which contribute to the treatment of cerebrovascular and cardiovascular diseases (Chen et al., 2016; Zhou et al., 2016). Recently, increasing research has been conducted to increase the content of SalA in *S. miltiorrhiza* (Xiao et al., 2009; Zhang et al., 2010). In this study, we reported that a low concentration of copper (5 μM) could promote the biosynthesis of SalA in *S. miltiorrhiza*. Then, we conducted comprehensive, integrated metabolomic and transcriptomic analyses to provide insights into the mechanisms underlying low copper-induced SalA biosynthesis. Genes-encoding SalAs biosynthetic enzymes (laccases and rosmarinate synthase) and transcription factors were identified as potential key factors involved in low copper-induced SalA biosynthesis.

### A Low Copper Concentration Promotes SalA Biosynthesis in *S. miltiorrhiza* Through Regulating Plant Growth and Photosynthesis

Our results revealed that the growth status and fresh weight of *S. miltiorrhiza* were significantly improved in the low copper concentration treatment compared with the control. The metabolites involved in carbon fixation in photosynthesis were significantly increased by the low-copper concentration treatment. Importantly, chlorophyll a, chlorophyll b, and total chlorophyll, which play an important role in photosynthesis (Baker, 2008), showed an increase in accumulation in low copper-treated plants. Photosynthesis contributes greatly to the biosynthesis of secondary metabolites in plants (Landi et al., 2020). A previous study has shown that copper played a role in photosynthesis through strengthening the photosynthetic capacity of plants (Douglas and Jaqueline, 2019). In our study, blue copper protein and basic blue protein, which are involved in promoting photosynthesis through acting as electron acceptors (Skyes, 1991), were upregulated in low copper-treated plants. Sal B and RA, the two most representative secondary metabolites of phenolic acids in *S. miltiorrhiza* (Zhao et al., 2015; Shi et al., 2016, 2019; Zhou et al., 2017; Huang et al., 2019), were significantly increased in *S. miltiorrhiza* treated with a low concentration of copper. These results suggest that low concentrations of copper induce the accumulation of SalAs might be through regulating photosynthesis related genes such as blue copper proteins in *S. miltiorrhiza*.

### Low Concentration of Copper Induces the Accumulation of SalAs by Activating Key Enzymes Involved in SalA Biosynthesis

Both tyrosine- and phenylalanine-derived pathways are the starting point for the biosynthesis of SalAs s (Hao et al., 2020). The data obtained in the transcriptomic analysis showed that rosmarinate synthase and laccase were upregulated in plants treated with a low copper concentration compared with the control. Rosmarinate synthase is the key enzyme in the pathway of SalAs biosynthesis in *S. miltiorrhiza* (Zhou et al., 2018). A previous study has reported that phenolic acids, such as rosmarinic acid and lithospermic acid B, were decreased in rosmarinate synthase-knockdown hairy root lines of *S. miltiorrhiza* (Zhou et al., 2018). However, overexpression of rosmarinate synthase resulted in a higher content of phenolic acids (up to over 3-fold) in transgenic lines compared with the control (Fu et al., 2020). Recently, scientists have demonstrated that laccase plays a critical role in the biosynthesis of Sal B. The laccase gene family in *S. bowleyana* has undergone expansion, and it might catalyze the oxidative reaction from RA to Sal B (Zheng et al., 2021). At present, 29 laccase candidates have been found in *S. miltiorrhiza*, and they contained three signature Cu-oxidase domains (Li et al., 2019b). It is known that laccases are copper-containing enzymes, and their catalytic activity requires the presence of copper ions (Arregui et al., 2019). Here, a core motif of a copper-response element (GTAC) and a metal response-element (TGCxCxC) were identified in the promoter region of laccase 20 and laccase 13. Overexpression and silencing assays demonstrated *SmLAC7* and *SmLAC20* played an important role in the biosynthesis of Sal B (Li et al., 2019b). In our study, laccase 20, laccase 13, laccase 7, and rosmarinate synthase were significantly upregulated in *S. miltiorrhiza* in the low-copper treatment compared with the control, suggesting that treatment with a low concentration of copper may promote the accumulation of SalAs *via* the regulation of laccase 20/13 and rosmarinate synthase.

In addition to the upregulation of laccase 20/13 and rosmarinate synthase, transcription factors, such as ethylene-responsive transcription factor (*ERF*), transcription factor MYBs, and basic helix-loop-helix transcription factor (*bHLH*), were also responsive to the low-copper treatment. The content of SalAs was increased in *SmERF115*-overexpressing hairy roots and was decreased in silencing lines (Sun et al., 2019). Previous studies have reported that *SmMYB1* was a positive activator that improved the accumulation of phenolic acids in *S. miltiorrhiza* (Deng et al., 2020b; Zhou et al., 2021). However, *SmMYB36* was reported to inhibit the accumulation of phenolic acids (Ding et al., 2017). For the *bHLH* transcription factor, the overexpression of *SmbHLH37* substantially decreased yields of Sal B (Du et al., 2018). Here, zinc finger protein ZAT10, Myb family transcription factor PHL5, ethylene-responsive transcription factor 5, and bZIP transcription factor 18 were significantly upregulated and coexpressed with laccase 20 under low concentration of copper treatment in *S. miltiorrhiza*. In our study, a core motif of a copper-response element (GTAC) and a metal-response element (TGCxCxC) were identified in the promoter region of transcription factors (zinc finger protein ZAT10, Zinc finger CCCH domain-containing protein 33, transcription factor MYB73, and transcription factor MYB33). In addition, MYB transcription factor-binding motifs were identified in promoter sequences of laccase 20 and laccase 13. In *Arabidopsis*, copper has been shown to induce expression changes of transcription factors, including bHLH, MYB, and ERF (Jakubowicz et al., 2010; Perea-García et al., 2010; Cai et al., 2021). Combined, these results suggest that transcription factors, such as ERF, MYB, and bHLH, play a role in copper-induced SalA biosynthesis in *S. miltiorrhiza*.

We systematically studied the effects of copper on SalA biosynthesis in *S. miltiorrhiza*. We identified a suitable copper ion concentration to promote the biosynthesis of SalA and provide a potential regulatory mechanism by identifying a series of copper-responsive genes. Our work provides an effective approach to induce SalAs and demonstrates a promising future for the metabolic regulation of *S. miltiorrhiza*.

## Data Availability Statement

The datasets presented in this study can be found in online repositories. The names of the repository/repositories and accession number(s) can be found here: https://www.ncbi.nlm.nih.gov/, PRJNA645746.

## Author Contributions

XY conceived and designed the research and revised the manuscript. YX, WS, XW, and JD analyzed the data and drafted the manuscript. WS and XW performed the experiments. All authors reviewed and approved the final manuscript.

## Funding

This work was supported by the National Natural Science Foundation of China (Grant No. 81803654).

## Conflict of Interest

The authors declare that the research was conducted in the absence of any commercial or financial relationships that could be construed as a potential conflict of interest.

## Publisher's Note

All claims expressed in this article are solely those of the authors and do not necessarily represent those of their affiliated organizations, or those of the publisher, the editors and the reviewers. Any product that may be evaluated in this article, or claim that may be made by its manufacturer, is not guaranteed or endorsed by the publisher.
