# Supplementary material for: Integrative Omics Analyses Reveal the Effects of Copper Ions on Salvianolic Acid Biosynthesis
Source: Front Plant Sci. 2021 Oct 21;12:746117. doi: 10.3389/fpls.2021.746117 (PMC8567050; doi:10.3389/fpls.2021.746117)
Supplement: Supplementary file 1 [file Presentation_1.PPTX]

## Slide 1
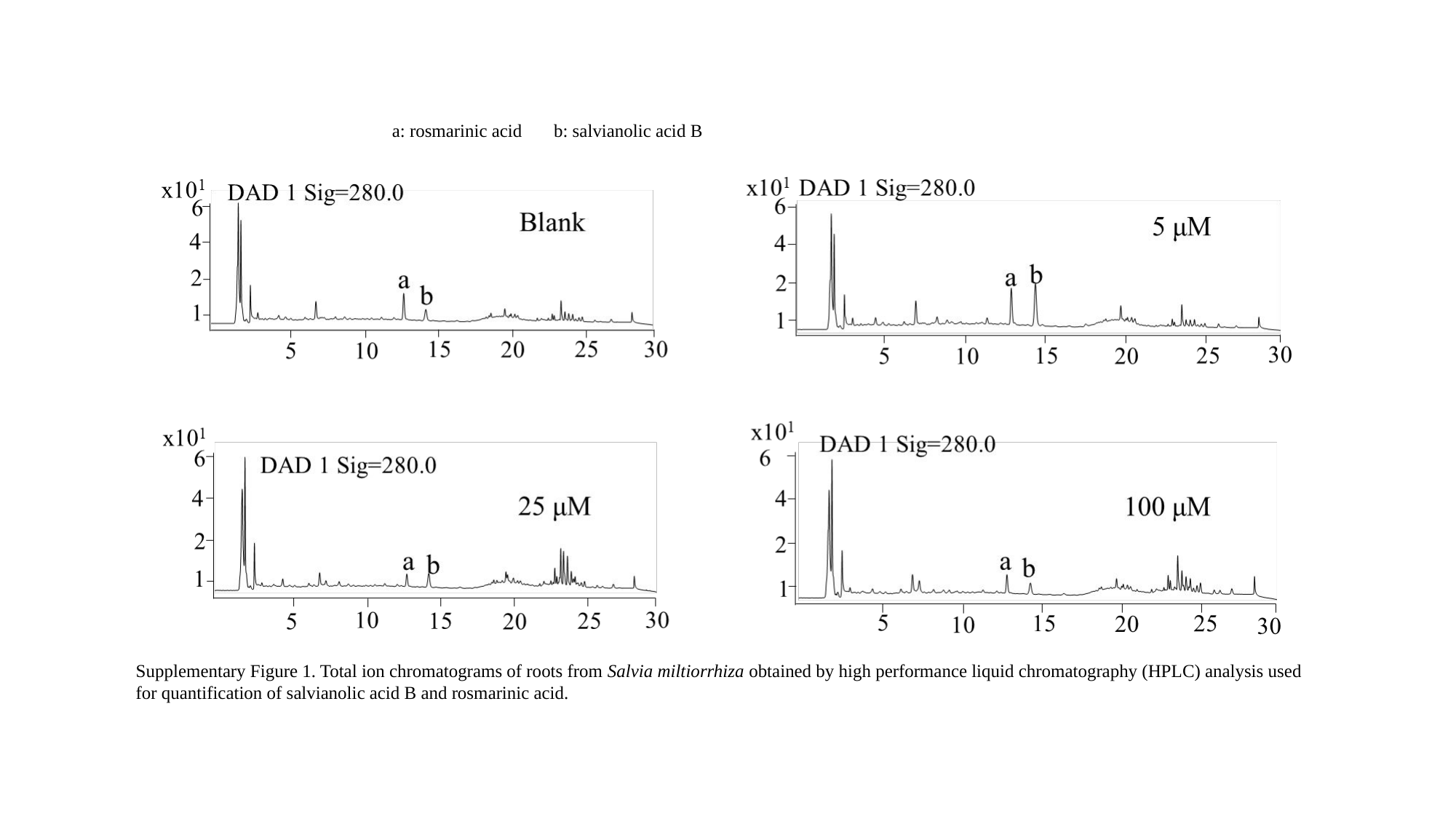

a: rosmarinic acid b: salvianolic acid B
Supplementary Figure 1. Total ion chromatograms of roots from Salvia miltiorrhiza obtained by high performance liquid chromatography (HPLC) analysis used for quantification of salvianolic acid B and rosmarinic acid.

## Slide 2
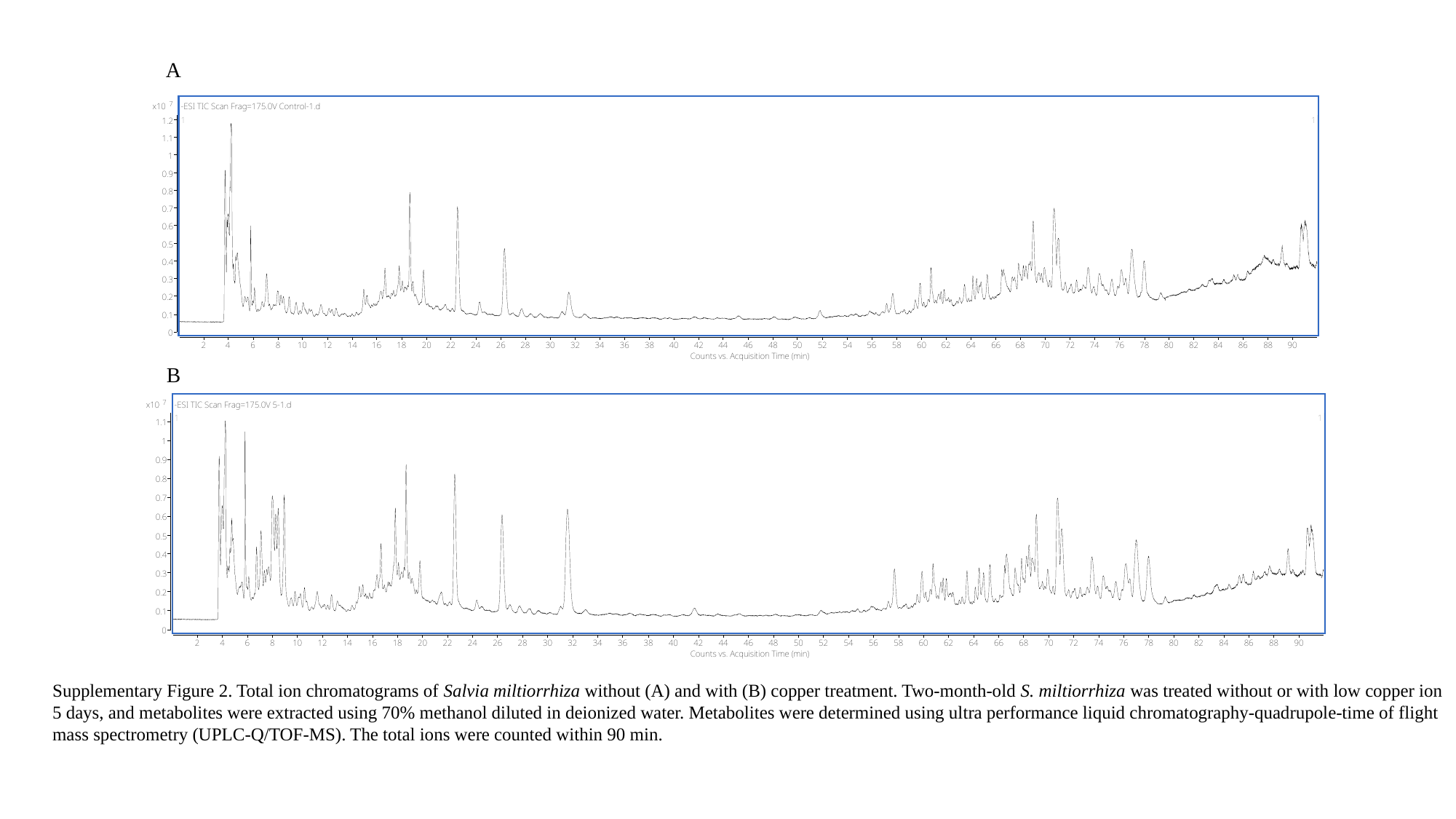

A
B
Supplementary Figure 2. Total ion chromatograms of Salvia miltiorrhiza without (A) and with (B) copper treatment. Two-month-old S. miltiorrhiza was treated without or with low copper ion 5 days, and metabolites were extracted using 70% methanol diluted in deionized water. Metabolites were determined using ultra performance liquid chromatography-quadrupole-time of flight mass spectrometry (UPLC-Q/TOF-MS). The total ions were counted within 90 min.

## Slide 3
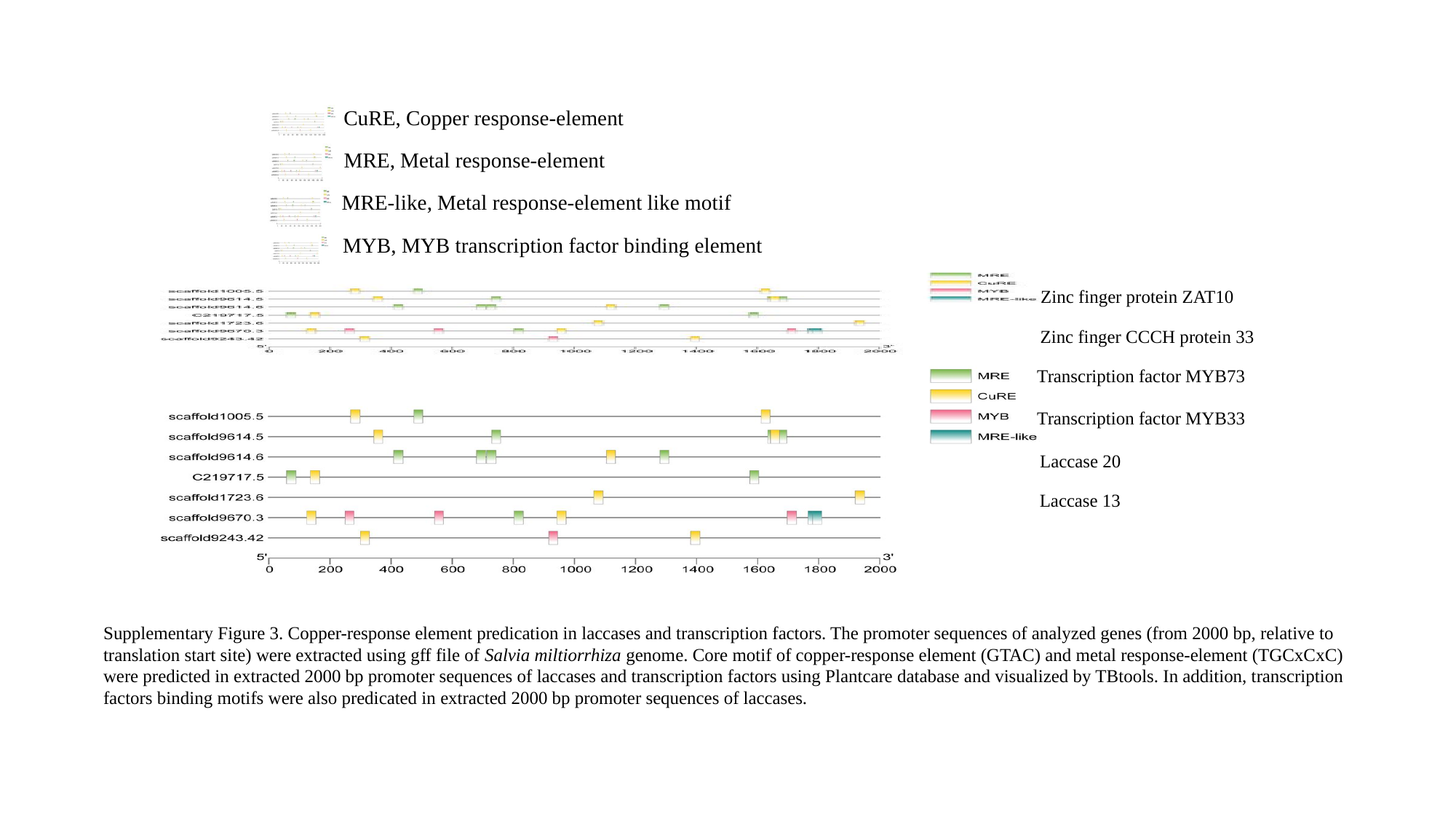

CuRE, Copper response-element
MRE, Metal response-element
MRE-like, Metal response-element like motif
MYB, MYB transcription factor binding element
Zinc finger protein ZAT10
Zinc finger CCCH protein 33
Transcription factor MYB73
Transcription factor MYB33
Laccase 20
Laccase 13
Supplementary Figure 3. Copper-response element predication in laccases and transcription factors. The promoter sequences of analyzed genes (from 2000 bp, relative to translation start site) were extracted using gff file of Salvia miltiorrhiza genome. Core motif of copper-response element (GTAC) and metal response-element (TGCxCxC) were predicted in extracted 2000 bp promoter sequences of laccases and transcription factors using Plantcare database and visualized by TBtools. In addition, transcription factors binding motifs were also predicated in extracted 2000 bp promoter sequences of laccases.
